# Women, Infants and Children (WIC) Participation and Breastfeeding Behavior: Comparing Foreign‐Born to US‐Born Mothers

**DOI:** 10.1111/birt.70081

**Published:** 2026-05-29

**Authors:** Angela G. Campbell

**Affiliations:** ^1^ Department of Applied Health Science, School of Public Health Indiana University Bloomington Indiana USA

**Keywords:** breastfeed, immigration, infant nutrition, WIC

## Abstract

**Background:**

This cross‐sectional study compared rates of breastfeeding initiation and exclusive breastfeeding among foreign‐born mothers who utilized the Women, Infants and Children (WIC) program with foreign‐born non‐participants and US‐born WIC participants.

**Methods:**

Sample children from the nationally representative dataset, the National Survey of Children's Health 2016–2022, ages 6 months to 1 year were included. Descriptive statistics and logistic regressions were executed for analysis.

**Results:**

91.8% of foreign‐born women who utilized WIC initiated breastfeeding and 34.8% exclusively breastfed for 6 months. By comparison, 68.2% of US‐born women who utilized WIC initiated breastfeeding and 10.4% exclusively breastfed for 6 months. Foreign‐born women who utilized WIC had 4.04 times the odds [95% CI (1.49, 10.97)] of initiating breastfeeding relative to foreign‐born women who did not utilize WIC. Foreign‐born women who utilized WIC also had 7.42 times the odds [95% CI (3.22, 17.11)] of initiating breastfeeding and 3.47 times the odds [95% CI (1.52, 7.95)] of exclusive breastfeeding for 6 months relative to US‐born women who utilized WIC (adjusted for household federal poverty level, preterm birth, birth order of sample child, sample child's race and mother's age, education, and marital status).

**Conclusion:**

Foreign‐born women who utilized WIC had increased breastfeeding behavior relative to both foreign‐born women who did not utilize WIC and US‐born women who utilized WIC. This suggests that, rather than WIC contributing to lower breastfeeding rates, there is an unobserved characteristic that is correlated with lower breastfeeding rates among US‐born women who utilize WIC that also encourages selection into the program.

## 1 Introduction

Breastfeeding is supported by the World Health Organization [1], the American Academy of Pediatrics [2], and the United States Centers for Disease Control and Prevention [3] as the preferred method of feeding infants. This support, alongside additional encouragement from goal setting initiatives such as Healthy People 2020 [4], has allowed for considerable growth in breastfeeding rates in the United States. We have improved from 74% [4] of women initiating breastfeeding in 2006 to 83.2% [5] of women initiating in 2019. We have also improved from 43.5% [4] of women engaging in any breastfeeding for 6 months in 2006 to 55.8% [5] in 2019. This progress should be celebrated, but there is still room for improvement. Healthy People 2030 aims for 42.4% of women to be exclusively breastfeeding by 2030 [6].

The reasons for the continued focus on breastfeeding are clear. Some of the health benefits to infants include: a lower risk of developing allergies [7], and asthma [7], for those who are ever breastfed and reduced risk of otitis media [8] and respiratory tract infections [2] among infants who are exclusively breastfed for 6 months. Women who breastfeed also benefit. Some of the health benefits include a reduced risk of breast cancer [9], among women who initiate breastfeeding and who breastfeed for extended durations and improved postpartum weight loss [10] among women who engage in extended breastfeeding. Due to these demonstrated benefits, breastfeeding is strongly supported by Women Infants and Children (WIC); a supplemental nutrition program for low income families [11].

WIC provides food, nutrition education and health referrals for pregnant and postpartum women, as well as infants and children up to age 5 [11]. Postpartum women are eligible to receive WIC nutritional assistance for up to 6 months if they are not breastfeeding, and up to 1 year if they are breastfeeding [12]. It is estimated that WIC provided assistance for 39% of all births in the United States in 2022 [12]. It has been associated with reduced food insecurity [13, 14] and increased infant birth weights for beneficiaries [15].

Unfortunately, WIC participation has also been associated with reduced rates of breastfeeding relative to WIC non‐participants [16, 17, 18] and is sometimes pejoratively labeled a “formula provider.” [19] Given WIC's active promotion of breastfeeding as the optimal form of nutrition for infants [11] it is unclear why WIC participants continue to have reduced odds of breastfeeding. One of the difficulties of assessing the relationship between WIC utilization and breastfeeding is that it is difficult to separate the impact of being low‐income on breastfeeding from the impact of WIC, since only low‐income women are eligible for the program [11]. Low‐income women have reduced odds of initiating breastfeeding relative to higher income women due to an array of sociodemographic disadvantages such as lower rates of marriage, lower education, and higher rates of preterm births [20, 21]. However, even studies examining breastfeeding behaviors solely among WIC eligible women found that the women who opted into WIC had lower breastfeeding initiation rates [22] and shorter duration of breastfeeding [23]. On the other hand, at least one study using propensity score matching found that there was no relationship between WIC utilization and breastfeeding [24], so it is possible that the reduced breastfeeding behavior seen among women who enroll in WIC is due to self‐selection rather than a failing of the WIC program in terms of breastfeeding education and promotion. One way to examine this hypothesis is to look at the breastfeeding rates of foreign‐born women who participate in WIC and may be less aware of WIC's reputation for formula provision [19].

Foreign‐born women who immigrate to developed countries have historically had high rates of breastfeeding relative to their native‐born peers [25, 26]. In the United States, the majority of foreign‐born mothers are of Hispanic descent [27] and foreign‐born Hispanic women have higher odds of initiating breastfeeding and breastfeed for longer than native‐born Hispanic women [18, 28]. In fact, in the United States, the three largest foreign‐born sociodemographic groups: non‐Hispanic (NH) White, foreign‐born NH Black, and foreign‐born Hispanic women all have increased odds of any breastfeeding for 6 months relative to native‐born White women [29]. Further, there are no citizenship restrictions that would bar foreign‐born women from participating in WIC [30] and participation in the WIC program does not count negatively toward a green card or full citizenship application [30]. To qualify for WIC, a pregnant woman must simply reside in the state in which she is applying, meet the income requirements, and be determined to be at nutritional risk [12]. The high breastfeeding rates of foreign‐born women combined with the fact that they are equally as eligible for WIC benefits as US‐born women make them an ideal comparison group. If the WIC program has deleterious impacts on breastfeeding, then analytic models should show decreased breastfeeding behaviors among foreign‐born women who utilize WIC.

This study aimed to compare breastfeeding rates among foreign‐born WIC participants, foreign‐born non‐participants, US‐born WIC participants, and US‐born non‐participants. First, differences between groups that may impact breastfeeding rates were compared. Next, the relationship between WIC utilization and breastfeeding behavior in models stratified by foreign‐born status versus US‐born women was examined. Given the high rates of breastfeeding among all foreign‐born women in the United States, I hypothesized that foreign‐born women who utilize WIC would not have significantly different breastfeeding behaviors relative to their foreign‐born counterparts who do not utilize WIC. I also hypothesized that foreign‐born women who utilize WIC would have increased odds of ever breastfeeding, any breastfeeding for 6 months, and exclusive breastfeeding for 6 months compared to US‐born women.

## 2 Materials and Methods

### 2.1 Study Design and Sample

This is a cross‐sectional, observational study using a nationally representative cohort of infants. The National Survey of Children's Health (NSCH) is a nationally representative survey of all children (ages 0–17) in the United States (excluding those who are institutionalized) administered annually by internet and mail [31]. It was designed by the Health Resources and Services Administration's Maternal and Child Health Bureau. The U.S. Census Bureau is responsible for the dissemination of the NSCH and data collection [32]. All questions were answered by an adult representative of the sample child [31]. Additional information regarding the design and administration of the NSCH can be accessed elsewhere [31].

Sample years 2016–2022 of the NSCH were pooled to ensure an adequate sample of foreign‐born mothers. All data were downloaded in April of 2024 to ensure the utilization of NSCH's updated weights for race/ethnicity [33]. Missing data for race/ethnicity and federal poverty level were imputed by the NSCH survey administrators using hot‐deck and sequential regression imputation methods, respectively [34]. The analytic dataset was restricted to children who were 6 months to less than 1 year of age at the time of the survey. This was done because the question regarding WIC status was phrased as, “At any time DURING THE PAST 12 MONTHS, even for one month, did anyone in your family receive… Benefits from the Women, Infants, and Children (WIC) Program?” Responses were coded dichotomously as yes/no. Restricting the dataset by infant age ensured that respondents had utilized WIC at some point while pregnant with the sample child or during the sample child's infancy. The dataset was also restricted to sample children living with a female biological or adopted parent to allow for proper identification of mother's foreign‐born status. Households with two mothers were also dropped as there may be conflicting foreign‐born statuses. This left a total sample of 4640. Next, 288 observations with missing data on one of the study variables were dropped (6.2%)–resulting in a final sample of 4352.

### 2.2 Variable Construction and Analysis

Breastfeeding behaviors were defined as the following: (1) ever breastfed, (2) any breastfeeding for 6 months, (3) exclusive breastfeeding for 6 months, and (4) any breastfeeding for 1 year. To determine the answers for these variables, the sample child's representative was asked, “Was this child ever breastfed or fed breastmilk?” and could answer Yes or No. If the sample child's representative indicated yes to ever breastfeeding, they were then asked, “If yes, how old was this child when he or she COMPLETELY stopped breastfeeding or being fed breast milk?” The responses were recorded numerically in months. The survey respondents could also indicate that the child was still breastfeeding at the time of the survey. All responses indicating that the infant was still breastfeeding or stopped breastfeeding in month 6 of life or later were coded as breastfeeding for 6 months. To determine exclusive breastfeeding, the survey respondent was asked, “How old was this child when he or she was FIRST fed formula?” and “How old was this child when they were FIRST fed anything other than breast milk or formula? Include water, juice, cow's milk, sugar water, baby food, or anything else that your child might have been given.” In accordance with the CDC definition of exclusive breastfeeding, any intake of formula or other liquids/food prior to the 6 month mark meant that the child was coded as not exclusively breastfeeding [35].

Independent variables included: child's race/ethnicity and mother's foreign‐born status, mother's age, mother's marital status, mother's education, preterm birth, and household federal poverty level. The sample child was determined as having a foreign‐born mother if one of the sample adults included in the interview was identified as a female, biological or adopted parent who was born outside of the United States. Mother's education was categorized into the following groups: “Less than High School,” “High School Graduate,” “Vocational Degree or Some College,” “College Graduate,” “Graduate School Degree.” Mother's age was coded into the following categories: “18–20,” “21–24,” “25–29,” “30–34,” “35–39,” “40 plus.” Marital status is coded dichotomously. Preterm birth was determined by a question asking, “Was this child born more than 3 weeks before their due date?” Yes answers were coded as preterm. The survey did not ask about the race or ethnicity of adults in the household, therefore, only the sample child's race is included in the analysis. Race is coded as, “Non‐Hispanic White,” (NH White), “Non‐Hispanic Black,” (NH Black) “Non‐Hispanic Asian,” (NH Asian) “Non‐Hispanic Other” (NH Other) and “Hispanic.” “NH Other” includes all sample children coded as American Indian, Alaskan Native, Native Hawaiian, other Pacific Islander, other race, and two or more races. These control variables were chosen as they have demonstrated significant associations with breastfeeding initiation or duration in previous work [20, 36, 37].

Descriptive statistics and logistic models were executed with survey weights to be nationally representative in accordance with NSCH recommendations [34]. All logistic models were first executed as bivariate models (unadjusted) and then as multivariate models (adjusted). Adjusted models included all control variables listed in the previous paragraph. The models were run using multiple imputation commands to account for the multiply imputed federal poverty level variable calculated by the NSCH. No other variables were imputed. All analyses were executed using STATA 18.

## 3 Results

### 3.1 Sample Descriptives

Among mothers who utilized WIC, the sample was 25.3% Hispanic for US‐born respondents and 54.9% Hispanic for foreign‐born respondents (Table 1). Among mothers who did not use WIC, the sample was 16.3% Hispanic for US‐born respondents and 35.8% Hispanic for foreign‐born respondents. Among mothers who utilized WIC, the sample of US‐born women was 39.3% NH White compared to 9.9% NH White in the foreign‐born sample. Among women who did not utilize WIC, the US‐born sample was 70.8% NH White and the foreign‐born sample was 18.1%.

**TABLE 1:** Descriptives by WIC Use and Foreign‐Born Status.

Variables	US‐Born No WIC (*N* = 3031)	US‐Born utilized WIC (*N* = 720)	Foreign‐Born No WIC (*N* = 420)	Foreign‐Born utilized WIC (*N* = 181)	Total sample (*N* = 4352)
All data are presented as weighted percentages					
Sample Child race/ethnicity					
NH White	70.8	39.3	18.1	9.9	52.5
NH Black	3.7	22.0	11.2	26.7	10.8
NH Asian	2.3	2.8	24.1	7.4	5.1
NH Other	6.9	10.6	10.8	1.1	7.6
Hispanic	16.3	25.3	35.8	54.9	23.9
Mother's age					
18–24	6.7	32.2	5.9	6.0	12.5
25–29	22.5	28.8	17.1	25.3	23.7
30–34	42.3	27.1	42.8	23.5	37.1
35–39	23.5	7.9	25.8	42.0	21.9
40+	5.0	4.0	8.4	3.3	4.9
Mother's marital status					
Currently not married	11.4	64.2	6.9	25.4	24.5
Currently married	88.6	35.8	93.1	74.6	75.6
Mother's highest level of education					
< 12 years	2.7	14.3	7.7	38.3	9.2
High school graduate	8.1	35.2	9.1	17.3	15.3
Vocational/some college	23.9	41.8	15.5	25.6	27.3
College degree	37.4	6.7	33.1	15.3	27.8
Graduate school	28.0	2.0	34.7	3.5	20.4
Preterm birth					
No	90.9	83.3	93.7	90.5	89.4
Yes	9.1	16.7	6.3	9.5	10.6
Birth order of sample child					
One	37.8	31.4	39.8	17.8	34.7
Two	36.8	33.0	33.4	35.3	35.5
Three plus	25.4	35.5	26.9	46.9	29.9
Federal poverty line*					
< 100% FPL	4.6	50.5	11.7	44.8	19.7
100%–199% FPL	12.8	32.2	12.5	38.6	19.6
200%–399% FPL	31.5	15.1	30.8	12.6	25.9
400% + FPL	51.1	2.2	44.8	4.0	35

*Note:* Percentages may add to slightly more or less than 100% due to rounding.

* The weighted data for poverty is calculated using the multiply imputed data given to us by the NSCH (mi estimate commands were used in STATA).

The US‐born sample was younger in both the “no WIC utilization” population and the “WIC utilization” population, with 71.5% and 88.1% of the sample being under 35. In the foreign‐born samples, only 65.8% and 54.8% of the “no WIC utilization” and “WIC utilization” populations were under 35, respectively. In both the US‐born and foreign‐born samples, a larger percentage of mothers who utilized WIC were unmarried relative to mothers who did not utilize WIC. This discrepancy was much larger for the US‐born sample; however, with 88.6% of “no WIC utilization” mothers married and only 35.8% of “WIC utilization” mothers married. In the foreign‐born sample, 93.1% of “no WIC utilization” mothers were married, while 74.6% of “WIC utilization” mothers were married.

Both foreign and US‐born mothers who utilized WIC had higher rates of no high school graduation relative to their foreign and US‐born peers who did not utilize WIC. However, the percentage of foreign‐born mothers who utilized WIC who did not graduate high school (38.3%) was much higher than the percentage of US‐born mothers who utilized WIC (14.3%). Foreign‐born women who did not utilize WIC had the lowest percentage of preterm births (6.3%), followed by foreign‐born women who utilized WIC (9.5%), US‐born women who did not utilize WIC (9.1%) and finally, US‐born women who utilized WIC (16.7%). Foreign‐born women who utilized WIC had the largest proportion of women with a sample child who was the third child or more (46.9%), followed by US‐born women who utilized WIC (35.5%), foreign‐born women who did not utilize WIC (26.9%) and US‐born women who did not utilize WIC (25.4%). In both the foreign‐born and US‐born populations, the majority of mothers who utilized WIC lived below 200% of the federal poverty line and the majority of mothers who did not utilize WIC lived at 200% of the federal poverty line or greater.

### 3.2 The Relationship Between WIC and Breastfeeding Behaviors in Models Stratified by Foreign‐Born Status

Foreign‐born women who utilized WIC had the highest rates of ever breastfeeding, and exclusive breastfeeding for 6 months compared to all other groups (Figure 1). Among foreign‐born women who utilized WIC, 91.8% ever breastfed, 61.7% engaged in any breastfeeding for 6 months and 34.8% exclusively breastfed for 6 months. In comparison, among US‐born mothers who did not utilize WIC, 91.2% ever breastfed, 64.6% engaged in any breastfeeding for 6 months and 26.2% exclusively breastfed for 6 months. US‐born mothers who utilized WIC have the lowest rates of ever breastfeeding (68.2%), any breastfeeding for 6 months (30.2%) and exclusive breastfeeding for 6 months (10.4%) compared to all other groups.

**FIGURE 1:**
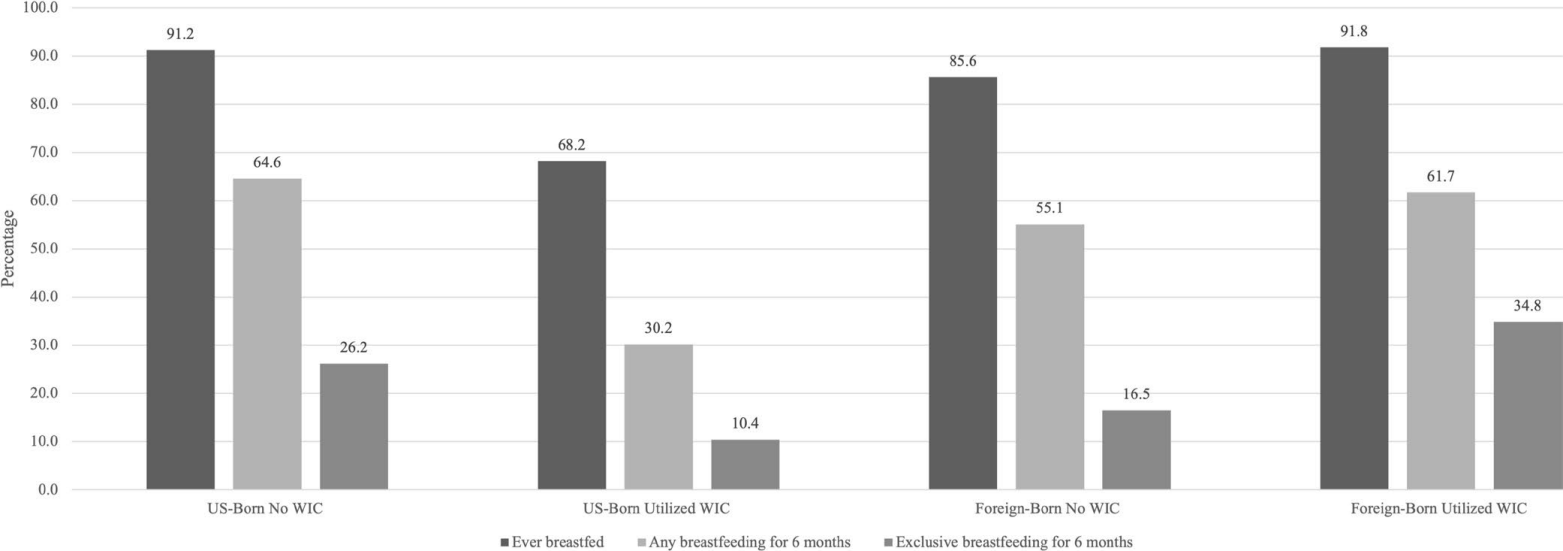
Breastfeeding Rates by Foreign‐born and WIC Utilization Status.

Table 2 examines the relationship of WIC with breastfeeding behaviors in a sample stratified by foreign‐born status. In the unadjusted models, US‐born mothers who utilized WIC in the past 12 months had significantly reduced odds of ever‐breastfeeding, any breastfeeding for 6 months and exclusive breastfeeding for 6 months. When adjusted for confounders, US‐born mothers who utilized WIC in the past 12 months had 0.43 times the odds of ever breastfeeding [OR = 0.43 95% CI (0.24, 0.79)], which is a 57% reduction in odds (*p* < 0.01). They also had 0.52 times the odds of any breastfeeding at 6 months [OR = 0.52 95% CI (0.35, 0.78)], which is a 48% reduction in odds (*p* < 0.01). There were no significant differences in the odds of exclusive breastfeeding for 6 months in the adjusted model. Among the foreign‐born sample, the unadjusted models showed that mothers who utilized WIC in the past 12 months had significantly increased odds of exclusive breastfeeding for 6 months. When adjusted for confounders, foreign‐born mothers who utilized WIC in the past 12 months had 4 times the odds of ever breastfeeding [OR = 4.04 95% CI (1.49, 10.97)] (*p* < 0.01), but there were no significant differences in the odds of any breastfeeding for 6 months or exclusive breastfeeding for 6 months.

**TABLE 2:** Logistic regression examining Odds of Breastfeeding Stratified by Nativity Status.

	Mother US‐Born (*N* = 3751)											
	Ever breastfed				Any breastfeeding for 6 months				Exclusive breastfeeding for 6 months			
	Unadjusted		Adjusted		Unadjusted		Adjusted		Unadjusted		Adjusted	
	OR	95% CI	OR	95% CI	OR	95% CI	OR	95% CI	OR	95% CI	OR	95% CI
Mother Utilized WIC In the Past 12 Months	0.21***	(0.14, 0.31)	0.43**	(0.24, 0.79)	0.24***	(0.17, 0.34)	0.52**	(0.35, 0.78)	0.33***	(0.20, 0.54)	0.74	(0.42, 1.29)

	Mother Foreign‐Born (*N* = 601)											
	Ever Breastfed				Any Breastfeeding for 6 Months				Exclusive Breastfeeding for 6 Months			
	Unadjusted		Adjusted		Unadjusted		Adjusted		Unadjusted		Adjusted	
	OR	95% CI	OR	95% CI	OR	95% CI	OR	95% CI	OR	95% CI	OR	95% CI
Mother Utilized WIC In the Past 12 Months	1.88	(0.71, 5.01)	4.04**	(1.49, 10.97)	1.31	(0.65, 2.66)	1.61	(0.71, 3.67)	2.69*	(1.06, 6.80)	1.5	(0.60, 3.75)

*Note:* Adjusted models control for year of survey, sample child's race, mother's age, mother's education, sample child's birth order, mother's marital status, preterm birth, and household poverty level.

* *p* < 0.05.

** *p* < 0.01.

*** *p* < 0.001.

### 3.3 The Relationship Between WIC, Foreign‐Born Status and Breastfeeding in the Full Sample

The impact of WIC participation was examined in the full sample in Table 3. In the adjusted models, foreign‐born mothers who utilized WIC had 7.42 times the odds [OR = 7.42 95% CI (3.22, 17.11)] of ever breastfeeding compared to US‐born women who utilized WIC, but foreign‐born women who did not utilize WIC were not significantly different. US‐born women who did not utilize WIC had 2.07 times the odds [OR = 2.07 95% CI (1.15, 3.72)] of ever breastfeeding compared to US‐born women who utilized WIC. Relative to US‐born women who utilized WIC, US‐born women who did not utilize WIC had 1.88 times the odds of any breastfeeding for 6 months [OR = 1.88 95% CI (1.22, 2.89)] and foreign‐born women who utilized WIC had 2.7 times the odds of any breastfeeding for 6 months [OR = 2.70 95% CI (1.44, 5.05)]. Foreign‐born women who did not utilize WIC did not have significantly different odds of any breastfeeding for 6 months compared to US‐born women who utilized WIC when adjusted for confounders. Finally, foreign‐born women who utilized WIC had 3.47 times the odds of exclusive breastfeeding for 6 months [OR = 3.47 95% CI (1.52, 7.95)] compared to US‐born women who utilized WIC. In the adjusted models, the odds of exclusive breastfeeding for 6 months were not significantly different for foreign‐born women who did not utilize WIC or US‐born women who did not utilize WIC compared to US‐born women who utilized WIC.

**TABLE 3:** Logistic regression examining Odds of Breastfeeding by Nativity and WIC Utilization Status (*N* = 4352).

	Ever breastfed				Any breastfeeding for 6 months				Exclusive breastfeeding for 6 months			
	Unadjusted		Adjusted		Unadjusted		Adjusted		Unadjusted		Adjusted	
	OR	95% CI	OR	95% CI	OR	95% CI	OR	95% CI	OR	95% CI	OR	95% CI
US‐Born No WIC	4.83***	(3.28, 7.13)	2.07*	(1.15, 3.72)	4.22***	(2.97, 6.01)	1.88**	(1.22, 2.89)	3.05***	(1.84, 5.04)	1.82	(0.96, 3.43)
US‐Born Utilized WIC	ref		ref		ref		ref		ref		ref	
Foreign‐Born No WIC	2.76*	(1.19, 6.43)	1.63	(0.69, 3.89)	2.84***	(1.68, 4.82)	1.15	(0.61, 2.18)	1.70	(0.91, 3.20)	1.02	(0.39, 2.65)
Foreign‐Born Utilized WIC	5.21***	(2.65, 10.22)	7.42***	(3.22, 17.11)	3.72***	(1.89, 7.33)	2.70**	(1.44, 5.05)	4.58**	(1.74, 12.08)	3.47**	(1.52, 7.95)

*Note:* Adjusted models control for year of survey, sample child's race, mother's age, mother's education, sample child's birth order, mother's marital status, preterm birth, and household poverty level.

* *p* < 0.05.

** *p* < 0.01.

*** *p* < 0.001.

## 4 Discussion

This study is the first study to examine the breastfeeding behaviors of foreign‐born women who utilize WIC in the United States. Previous literature has established that, on average, women utilizing WIC are less likely to breastfeed [16, 17, 18] and the results for the US‐born sample align with these findings. However, contrary to the hypothesis that there would be no differences in breastfeeding behaviors by WIC utilization among foreign‐born women, I found that foreign‐born women who utilized WIC had significantly higher breastfeeding initiation rates relative to foreign‐born women who did not utilize WIC. They also had breastfeeding initiation and any breastfeeding rates that were comparable to US‐born women who did not use WIC and had significantly increased odds of ever breastfeeding, any breastfeeding for 6 months, and exclusive breastfeeding for 6 months relative to US‐born women who utilized WIC, which aligned with our hypothesis.

Foreign‐born women who migrate to first world countries generally have higher breastfeeding initiation rates [26] and breastfeed for longer durations than native‐born women [26]. This is the first study to establish that, in the United States, WIC was positively associated with this behavior among foreign‐born women. Surprisingly, foreign‐born women on WIC had increased odds of initiating breastfeeding relative not only to US‐born women who utilized WIC, but also to foreign‐born women who did not participate in WIC.

Exclusive breastfeeding for 6 months was also very high among foreign‐born women who utilized WIC. Previous work has shown that foreign‐born women who migrate to developed countries are more likely to engage in extended breastfeeding, but not necessarily exclusive breastfeeding [26]. Postnatal breastfeeding behaviors have been linked with prenatal perceptions of WIC breastfeeding recommendations among WIC participants [38]. If foreign‐born women do not have preconceived notions regarding WIC recommendations, they may be more receptive to the breastfeeding education initiatives from WIC.

This study is not the first to find that WIC participation is linked with improved breastfeeding behaviors among a subgroup of participants. Our results align with previous work that found WIC participation was associated with increased odds of breastfeeding initiation among minority women [39, 40], especially those with less than a high school education [39]. Analyzing the influence of WIC on different demographics in the United States should be a high priority for researchers and policy makers who wish to accurately assess the impact of this program.

One critical limitation of this study is the inability to control for the foreign‐born mother's country of origin. On average, low‐ and middle‐income countries have higher rates of breastfeeding initiation and longer durations of breastfeeding relative to high‐income countries [41]. It is possible that WIC use is a proxy for country of origin among foreign‐born women, with mothers from low‐ and middle‐income countries being more likely to utilize WIC. Globally, it is estimated that about 48% of women breastfeed exclusively between the ages of 0–5 months [42]. Among mothers from Latin America and the Caribbean, the most common ethnic group in our foreign‐born WIC population, 43% breastfed exclusively for 0–5 months [42]. In this study's sample, 34.8% of foreign‐born mothers who utilized WIC breastfeed exclusively for at least 6 months. Our estimate is likely lower due to the increased time duration of our exclusive breastfeeding measure as many women may be introducing table foods after the fifth month [43]. However, it could also be lower due to differences in breastfeeding practices between women from different cultural backgrounds who migrate to the United States.

This study was not able to control for acculturation, but future work should also consider the possibility that acculturation within the foreign‐born sample could increase WIC enrollment among foreign‐born women who do not wish to breastfeed if they become aware of WIC's reputation as a “formula provider.” [19] Conversely, less acculturated women who enroll in WIC without preconceived ideas of WIC's purpose may be more receptive to its breastfeeding promotion efforts. Appendix SA and Appendix SB show the results of the analytic models when the sample is restricted to women who ever breastfed. In these models, all differences in breastfeeding duration by foreign‐born and WIC utilization status were explained by the control variables included in the adjusted models. This suggests that among women who initiate breastfeeding there are differences in breastfeeding duration, but that those differences are associated with sociodemographic factors rather than some other variable that is being proxied by WIC enrollment. Therefore, WIC status is serving as a proxy for the factors associated with the decision to initiate and operating in different directions depending on foreign‐born status. This could also be indirectly associated with any breastfeeding and exclusive breastfeeding duration as the same initiatives associated with breastfeeding initiation could be impacting other breastfeeding decisions for these women. Additional work is needed to explore this possibility.

Another important factor that affects extended breastfeeding is employment status [44, 45], but this dataset did not include information on employment for all years. Foreign‐born women on WIC may be less likely to be employed, and therefore, could have an increased likelihood of engaging in exclusive breastfeeding for extended periods due to not being separated from their infant. We also do not know if any of these women pumped breastmilk, which could also be an indicator of returning to work. Also, while there is a control for preterm birth in our analytic models, the NSCH data does not have information on the infant's exact gestational age at birth. Infants who were very preterm have more difficulty breastfeeding than infants who were moderately preterm [46].

This survey was not specifically designed to study the relationship of WIC utilization with breastfeeding. Therefore, the questions are vague regarding the timing of WIC utilization. The NSCH asked if WIC was utilized within the past 12 months, but we cannot pinpoint exactly when. The sample was limited to infants between the age of 6 months and 1 year so that we know WIC was utilized either during the mother's pregnancy or infant's first year of life, but it is impossible to be more precise. This also makes it difficult to interpret the relationship of household poverty level to WIC utilization as the household poverty level can vary greatly over the course of a year due to job attainment/loss, marriage/divorce or the birth/death of dependents [47]. Finally, two of the survey years included in this analysis were gathered during the Covid epidemic in 2020 and 2021 [48]. There was not a large enough sample size to examine time trends and determine if Covid impacted breastfeeding during this time. However, the survey was administered via mail and internet [31] so restrictions on in‐person visitation likely did not impact our sample and the response rates for 2020 and 2021 were similar to previous years [31, 34].

## 5 Conclusions

This study provides new evidence that WIC is associated with positive breastfeeding outcomes among foreign‐born women in the United States. These findings challenge preconceptions that WIC is associated with reduced breastfeeding among its beneficiaries [19] and support studies that found positive impacts of WIC among specific demographics [39, 40]. Additional work is needed to explore the factors driving breastfeeding behaviors among foreign‐born women utilizing WIC, as this study was not able to explore variables such as employment status in the post‐partum period. However, these results are an indication that WIC is not detrimental to breastfeeding among foreign‐born participants and could be beneficial. It also suggests that low breastfeeding rates seen among US‐born WIC participants may be due to sociodemographic factors or other omitted variables associated with WIC enrollment rather than the WIC program itself. Understanding the variables influencing breastfeeding success among foreign‐born women who utilize WIC may serve as a leverage point to expand this success to all women utilizing WIC, regardless of nativity status.

## Funding

This work was funded, in part, by the Health Excellence Achieved through Learning health system Research (HEAL‐R) Collaborative.

## Ethics Statement

This study was approved by Indiana University Institutional Review Board as not human subjects research (IRB Protocol #23134).

## Conflicts of Interest

The author declares no conflicts of interest.

## Supporting information


**Appendix SA:** Logistic regression examining Odds of Breastfeeding Stratified by Nativity Status—Limited to Women Who Initiated Breastfeeding.
**Appendix SB:** Logistic regression examining Odds of Breastfeeding by Nativity and WIC Utilization Status (*N* = 3878)–Limited to Women Who Initiated Breastfeeding.
