# Supplementary material for: Women, Infants and Children (WIC) Participation and Breastfeeding Behavior: Comparing Foreign‐Born to US‐Born Mothers
Source: Birth. 2026 May 29;53(4):731–9. doi: 10.1111/birt.70081 (PMC13728509; doi:10.1111/birt.70081)
Supplement: Supplementary file 1 [file BIRT-53-731-s001.docx]

| **Supplementary Appendix A. Logistic regression examining Odds of Breastfeeding Stratified by Nativity Status – Limited to Women Who Initiated Breastfeeding** | | | | | | | | |
| --- | --- | --- | --- | --- | --- | --- | --- | --- |
|  | **Mother US-Born (N=3,343)** | | | | | | | |
|  | Any Breastfeeding for 6 Months | | | | Exclusive Breastfeeding for 6 Months | | | |
|  | Unadjusted |  | Adjusted |  | Unadjusted |  | Adjusted |  |
|  | OR | 95% CI | OR | 95% CI | OR | 95% CI | OR | 95% CI |
| Mother Utilized WIC In the Past 12 Months | 0.32*** | (0.22, 0.48) | 0.61* | (0.38, 0.99) | 0.44** | (0.27, 0.75) | 0.90 | (0.51, 1.59) |
|  | **Mother Foreign-Born (N=535)** | | | | | | | |
|  | Any Breastfeeding for 6 Months | | | | Exclusive Breastfeeding for 6 Months | | | |
|  | Unadjusted | | Adjusted | | Unadjusted | | Adjusted | |
|  | OR | 95% CI | OR | 95% CI | OR | 95% CI | OR | 95% CI |
| Mother Utilized WIC In the Past 12 Months | 1.13 | (0.53, 2.39) | 0.97 | (0.38, 2.49) | 2.55 | (0.99, 6.54) | 0.96 | (0.36, 2.56) |
| *Adjusted models control for year of survey, sample child's race, mother's age, mother's education, sample child's birth order, mother's marital status, preterm birth and household poverty level | | | | | | | | |
| *p<.05, **p<.01, ***p<.001 | |  |  |  |  |  |  |  |

| **Supplementary Appendix B. Logistic regression examining Odds of Breastfeeding by Nativity and WIC Utilization Status (N=3,878) – Limited to Women Who Initiated Breastfeeding** | | | | | | | | |
| --- | --- | --- | --- | --- | --- | --- | --- | --- |
|  | Any Breastfeeding for 6 Months | | | | Exclusive Breastfeeding for 6 Months | | | |
|  | Unadjusted | | Adjusted | | Unadjusted | | Adjusted | |
|  | OR | 95% CI | OR | 95% CI | OR | 95% CI | OR | 95% CI |
| US-Born No WIC | 3.06*** | (2.09, 4.47) | 1.59 | (0.97, 2.98) | 2.23** | (1.33, 3.73) | 1.53 | (0.79, 2.95) |
| US-Born Utilized WIC | ref |  | ref |  | ref |  | ref |  |
| Foreign-Born No WIC | 2.28** | (1.32, 3.94) | 0.98 | (0.49, 1.99) | 1.33 | (0.70, 2.51) | 0.92 | (0.34, 2.47) |
| Foreign-Born Utilized WIC | 2.58* | (1.26, 5.30) | 1.42 | (0.73, 2.78) | 3.38* | (1.26, 9.08) | 2.29 | (1.00, 5.25) |
| *Adjusted models control for year of survey, sample child's race, mother's age, mother's education, sample child's birth order, mother's marital status, preterm birth and household poverty level | | | | | | | | |
| *p<.05, **p<.01, ***p<.001 | |  |  |  |  |  |  |  |
